# Expression and function of FcγRII on NK cells in rhesus macaques

**DOI:** 10.1186/1742-4690-9-S2-P38

**Published:** 2012-09-13

**Authors:** X He, H Peng, Z Luo, D Liu, H Liang, Y Shao

**Affiliations:** 1 National Center for AIDS/STD Control and Prevention, China CDC, Beijing, China

## Background

Natural Killer (NK) cells play a crucial role in ADCC response through FcγRIIIa (CD16), which is able to bind to Fc region of IgG displayed on target cells. The presence of another class of low-affinity receptor for IgG, called FcγRII(CD32), has also been reported in human. It raises the possibility of CD32 expression on NK cells in non-human primate models. Efforts to get a clearer understanding of the CD32 marker which might be related to ADCC function of NK cells in rhesus macaques will be helpful in evaluation of vaccines in non-human primate models.

## Methods

PBMCs were obtained from a total of 42 healthy macaques in the study and cellular immunology of NK cells was analyzed by flow cytometry. CD32 Blocking antibodies were added to PBMCs for FcγRII blockade assay.

## Results

The expression of CD32 was observed on macaque NK cells (Median, 1.45%; range, 0.96%-2.20%) and was negatively correlated with CD16 expression (P<0.001). The downregulation of CD16 after stimulation by antibody-coated target cells was viewed as an important indicator of NK-cell activation, for its strong positive relationship with CD107a, IFN-γ or TNF-α measured in ADCC assay (P<0.001 for all). Either the percentage or MFI of CD16 expression on NK cells decreased in the presence of CD32 blocking antibodies, especially in early stage of ADCC. Surprisingly, after CD32 blocking, the percentage of CD107a+ NK cells shifted from increase at the very beginning of ADCC activity (P<0.05) to decrease when NK cells approached the complete activation by antibody-coated target cells (P<0.01).

## Conclusion

CD32^+^ NK cells defined a novel NK-cell subset in macaques and we found that CD32 had dual function with a time-course manner in ADCC activity. CD32 may attenuate or slow down the activation of macaque NK cells at first, then up-modulate function of NK cells at the late stage of ADCC.

